# Candidate Gene Resequencing in a Large Bicuspid Aortic Valve-Associated Thoracic Aortic Aneurysm Cohort: *SMAD6* as an Important Contributor

**DOI:** 10.3389/fphys.2017.00400

**Published:** 2017-06-13

**Authors:** Elisabeth Gillis, Ajay A. Kumar, Ilse Luyckx, Christoph Preuss, Elyssa Cannaerts, Gerarda van de Beek, Björn Wieschendorf, Maaike Alaerts, Nikhita Bolar, Geert Vandeweyer, Josephina Meester, Florian Wünnemann, Russell A. Gould, Rustam Zhurayev, Dmytro Zerbino, Salah A. Mohamed, Seema Mital, Luc Mertens, Hanna M. Björck, Anders Franco-Cereceda, Andrew S. McCallion, Lut Van Laer, Judith M. A. Verhagen, Ingrid M. B. H. van de Laar, Marja W. Wessels, Emmanuel Messas, Guillaume Goudot, Michaela Nemcikova, Alice Krebsova, Marlies Kempers, Simone Salemink, Toon Duijnhouwer, Xavier Jeunemaitre, Juliette Albuisson, Per Eriksson, Gregor Andelfinger, Harry C. Dietz, Aline Verstraeten, Bart L. Loeys, Mibava Leducq Consortium

**Affiliations:** ^1^Faculty of Medicine and Health Sciences, Center of Medical Genetics, University of Antwerp and Antwerp University HospitalAntwerp, Belgium; ^2^Cardiovascular Genetics, Department of Pediatrics, CHU Sainte-Justine, Université de MontrealMontreal, QC, Canada; ^3^Department of Cardiac and Thoracic Vascular Surgery, University Hospital Schleswig-HolsteinLübeck, Germany; ^4^McKusick-Nathans Institute of Genetic Medicine, Johns Hopkins University School of MedicineBaltimore, MD, United States; ^5^Department of Clinical pathology, Lviv National Medical University after Danylo HalytskyLviv, Ukraine; ^6^Cardiovascular Research, SickKids University HospitalToronto, ON, Canada; ^7^Cardiovascular Medicine Unit, Department of Medicine, Karolinska InstituteStockholm, Sweden; ^8^Cardiothoracic Surgery Unit, Department of Molecular Medicine and Surgery, Karolinska InstituteStockholm, Sweden; ^9^Department of Clinical Genetics, Erasmus University Medical CenterRotterdam, Netherlands; ^10^Assistance Publique–Hôpitaux de Paris, Hôpital Européen Georges Pompidou; Université Paris Descartes, Paris Sorbonne Cité; Institut National de la Santé et de la Recherche Médicale, UMRSParis, France; ^11^Department of Biology and Medical Genetics, 2nd Faculty of Medicine-Charles University and Motol University HospitalPrague, Czechia; ^12^Institute of Clinical and Experimental MedicinePrague, Czechia; ^13^Department of Human Genetics, Radboud University Medical CentreNijmegen, Netherlands; ^14^Howard Hughes Medical InstituteBaltimore, MD, United States

**Keywords:** bicuspid aortic valve, thoracic aortic aneurysm, SMAD6, targeted gene panel, variant burden test

## Abstract

Bicuspid aortic valve (BAV) is the most common congenital heart defect. Although many BAV patients remain asymptomatic, at least 20% develop thoracic aortic aneurysm (TAA). Historically, BAV-related TAA was considered as a hemodynamic consequence of the valve defect. Multiple lines of evidence currently suggest that genetic determinants contribute to the pathogenesis of both BAV and TAA in affected individuals. Despite high heritability, only very few genes have been linked to BAV or BAV/TAA, such as *NOTCH1, SMAD6*, and *MAT2A*. Moreover, they only explain a minority of patients. Other candidate genes have been suggested based on the presence of BAV in knockout mouse models (e.g., *GATA5, NOS3*) or in syndromic (e.g., *TGFBR1/2, TGFB2/3*) or non-syndromic (e.g., *ACTA2*) TAA forms. We hypothesized that rare genetic variants in these genes may be enriched in patients presenting with both BAV and TAA. We performed targeted resequencing of 22 candidate genes using Haloplex target enrichment in a strictly defined BAV/TAA cohort (*n* = 441; BAV in addition to an aortic root or ascendens diameter ≥ 4.0 cm in adults, or a Z-score ≥ 3 in children) and in a collection of healthy controls with normal echocardiographic evaluation (*n* = 183). After additional burden analysis against the Exome Aggregation Consortium database, the strongest candidate susceptibility gene was *SMAD6* (*p* = 0.002), with 2.5% (*n* = 11) of BAV/TAA patients harboring causal variants, including two nonsense, one in-frame deletion and two frameshift mutations. All six missense mutations were located in the functionally important MH1 and MH2 domains. In conclusion, we report a significant contribution of *SMAD6* mutations to the etiology of the BAV/TAA phenotype.

## Introduction

With a prevalence of 1–2% in the general population, bicuspid aortic valve (BAV) is the most common congenital heart defect. It has a 3:1 male preponderance and is characterized by an aortic valve with two cusps instead of the normal three. BAV often coincides with aortic manifestations such as coarctation of the aorta and thoracic aortic aneurysm (TAA) (Verstraeten et al., 2016). The latter can lead to lethal dissections if left untreated. Although first described over 400 years ago and high heritability (89%) (Cripe et al., 2004), the genetic etiology of BAV, with or without TAA, remains largely elusive. It was initially suggested that TAA results from altered blood flow dynamics imposed by the abnormal bicuspid valve. Changes in shear stress were presumed to weaken the aortic wall, resulting in dilatation and rupture. At present, common genetic risk factors for BAV and TAA are proposed (Hinton, 2012), based on the following observations: (i) the aortic valve and the aorta share common embryologic origins [i.e., the cardiac neural crest (CNC) and the second heart field] (Martin et al., 2015), (ii) family members of BAV/TAA probands show TAA without valve abnormalities and/or BAV without aneurysmal disease (Loscalzo et al., 2007), and (iii) TAA formation in BAV probands that previously underwent valve replacement has been reported (Braverman et al., 2005).

Transmission of BAV/TAA mostly complies with an autosomal dominant inheritance pattern, displaying reduced penetrance and variable expressivity (Clementi et al., 1996; Huntington et al., 1997). Few genes have been robustly linked to the BAV phenotype to date. *NOTCH1* is often considered the sole established BAV gene, either as an isolated finding or in association with early onset valve calcification, TAA, or other left-sided heart defects (Mohamed et al., 1797; Garg et al., 2005; McKellar et al., 2007; Foffa et al., 2013; Kent et al., 2013; Bonachea et al., 2014; Freylikhman et al., 2014; Kerstjens-Frederikse et al., 2016). *SMAD6* (Tan et al., 2012) and *MAT2A* (Guo et al., 2015) have also been implicated in BAV, but only in a very limited number of patients. A dozen candidate genes emanated from knockout mouse models with increased BAV occurrence (Biben et al., 2000; Lee et al., 2000; Laforest and Nemer, 2011; Laforest et al., 2011; Thomas et al., 2012; Mommersteeg et al., 2015; Quintero-Rivera et al., 2015). The prevalence of BAV in these knockout models is often low (range: 2–42% in single knockouts) (Table 1), probably due to reduced penetrance and/or activation of compensatory mechanisms. Mutations in some syndromic (Attias et al., 2009; Callewaert et al., 2011; Lindsay et al., 2012; Nistri et al., 2012; van de Laar et al., 2012; Pepe et al., 2014) or non-syndromic (Guo et al., 2007) TAA genes also associate with increased BAV occurrence (Table 1).

**Table 1:** Genes included in the targeted gene panel and the criteria on which their selection was based.

**Context**	**Gene**	**Incidence**	**References**
BAV in humans	*NOTCH1*	Mutations found in 27 BAV patients	Mohamed et al., 1797; Garg et al., 2005; McKellar et al., 2007; Foffa et al., 2013; Kent et al., 2013; Bonachea et al., 2014; Freylikhman et al., 2014; Kerstjens-Frederikse et al., 2016
	*SMAD6*	Mutations found in 2 BAV patients	Tan et al., 2012
	*MAT2A*	Mutations found in 1 BAV patient	Guo et al., 2015
BAV in mice	*ACVR1*	BAV in 78–83% of *Alk2^*FXKO*^/Gata5^−*Cre*+^* mice	Thomas et al., 2012
	*GATA4*	BAV in 43% of *Gata4^+/−^*;*Gata5^+/−^* mice	Laforest and Nemer, 2011
	*GATA5*	BAV in 25% of *Gata5^−/−^* mice	Laforest et al., 2011
	*GATA6*	BAV in 25% *Gata5^+/−^;Gata6^+/−^* mice	Laforest and Nemer, 2011
	*MATR3*	BAV in 12% in *Matr3^+/−^* mice	Quintero-Rivera et al., 2015
	*NKX2-5*	BAV in 2–20% of *Nkx2-5*^+/^*^−^* mice	Biben et al., 2000
	*NOS3*	BAV in 42% of *Nos3^−/−^* mice	Lee et al., 2000
	*ROBO1*	BAV in 100% of *Robo1^−/−^*;*Robo2^−/−^* mice	Mommersteeg et al., 2015
	*ROBO2*	BAV in 100% of *Robo1^−/−^*;*Robo2^−/−^* mice	Mommersteeg et al., 2015
BAV in (non)syndromic TAA cases	*FBN1*	Occasional BAV in Marfan syndrome	Attias et al., 2009; Nistri et al., 2012; Pepe et al., 2014
	*ACTA2*	7% BAV in non-syndromic TAA	Guo et al., 2007
	*ELN*	Occasional BAV in cutis laxa	Callewaert et al., 2011
	*FLNA*	Occasional BAV in X-linked valve disease	Jefferies et al., 2010
	*MYH11*	Occasional BAV in non-syndromic TAA	Personal observation
	*SMAD3*	3–11% BAV in Loeys-Dietz syndrome	van de Laar et al., 2012
	*TGFB2*	8–13% BAV in Loeys-Dietz syndrome	Lindsay et al., 2012
	*TGFB3*	4% BAV in Loeys-Dietz syndrome	Personal observation
	*TGFBR1*	8–12% BAV in Loeys-Dietz syndrome	Personal observation
	*TGFBR2*	8–12% BAV in Loeys-Dietz syndrome	Personal observation

*BAV, Bicuspid aortic valve; TAA, Thoracic aortic aneurysm*.

To date, no major BAV/TAA gene has emerged. The described genes have been associated with BAV, but their contribution to the etiology of BAV/TAA has never been examined systematically. Here, we evaluate this contribution in 22 BAV-associated genes (Table 1) using a targeted gene panel and variant burden approach.

## Materials and methods

### Study cohort

Genomic DNA (gDNA) of 441 BAV/TAA patients was collected through a collaborative effort involving 8 different centers (Supplementary Table 1). Patients were selected based on the presence of BAV and either an aortic diameter at the sinus of Valsalva or the ascending aorta of at least 4.0 cm in adults, or a Z-score exceeding 3 in children. Aortic diameter dimensions were determined using echocardiography, computed tomography or magnetic resonance imaging. A positive family history was defined as having at least one first- or second-degree relative with BAV and/or TAA. Control gDNA was obtained from 183 cancer patients who presented at the SickKids Hospital, Toronto, Canada. None of the controls showed structural heart disease upon examination with echocardiography. All study participants or their legal guardians gave informed consent at the respective sample-contributing centers.

### Targeted enrichment

Genes (*n* = 22) were selected for targeted resequencing based on the following criteria: (i) mutations occur in human BAV cases (*n* = 3), (ii) knockout mouse models present with incomplete penetrance of BAV (*n* = 9), and (iii) occasional or increased BAV manifestation occurs in patients with mutations in known TAA genes (*n* = 10) (Table 1). Enrichment of all exons of these candidate genes, including ±10 nucleotides of adjacent intronic sequence, was performed with a custom Haloplex target enrichment kit per instructions of the manufacturer (Agilent Technologies, USA). Probe design covered a theoretical 99.7% of the complete target region (560 kb). Pooled samples were sequenced either on a HiSeq 2500 (Illumina, USA) with 2 × 150 bp reads or on a HiSeq 1500 (Illumina, USA) with 2 × 100 bp reads.

### Data analysis and filtering

The raw data were processed using an in-house-developed Galaxy-based pipeline, followed by variant calling with the Genome Analysis Toolkit Unified Genotyper (DePristo et al., 2011). Variants were subsequently annotated and filtered with the in-house developed database VariantDB (Vandeweyer et al., 2014), which uses ANNOVAR. Heterozygous coding or splice site (±2 bp from exon-intron boundaries for nucleotide substitution, and ±5 bp for multi-bp deletions or insertions) variants with an allelic balance between 0.25 and 0.85 (*FLNA* in males: 0.75–1) and a minimum coverage of 10 reads were selected. Finally, we included variants that fitted within at least one of the following three categories; unique variants [absent in the Exome Aggregation Consortium (ExAC) database (Lek et al., 2016)], variants with an ExAC Minor Allele Frequency (MAF) lower than 0.01% or variants with an ExAC MAF between 0.01% and 0.1% that had a Combined Annotation Dependent Depletion (CADD) (Kircher et al., 2014) score above 20. All splice region variants underwent splice site effect prediction using ALAMUT (Interactive Biosoftware, France). Synonymous variants outside of splicing regions were not taken into account.

The ExAC database was used as an independent control dataset. The raw data of variants (~all ExAC datasets) fulfilling ExAC's quality control parameters (“PASS”) were extracted from the offline version of ExAC v0.3.1. Since the ExAC variants were annotated using VEP, whereas our patient variant annotation was ANNOVAR-based, we re-annotated the ExAC variants with ANNOVAR. The same variant filtering strategy as described for the patient cohort was subsequently applied. For each selected ExAC variant, the allele frequency was determined by computing the ratio of the Mutant Allele Count (mAC) and Total Allele Count (tAC). Next, we re-scaled each variant's mAC by multiplying its computed allele frequency by its respective tAC_Adj, i.e., the tAC average of all variants in that specific gene. Finally, the variant counts for each panel gene were obtained by summing up the re-scaled mACs.

### Validation by sanger sequencing

Variants discussed in the results section were confirmed with Sanger sequencing. Primers were designed using Primer3 software (Untergasser et al., 2012) v4.0.0 and polymerase chain reaction (PCR) products were purified with Calf Intestinal Alkaline Phosphatase (Sigma-Aldrich, USA). Sequencing reactions were performed using the BigDye Terminator Cycle Sequencing kit (Applied Biosystems, Life Technologies, USA), followed by capillary electrophoresis on an ABI3130XL (Applied Biosystems, Life Technologies, USA). The obtained sequences were analyzed with CLC DNA Workbench v5.0.2 (CLC bio, Denmark).

### Segregation analysis

When family members were available, Sanger sequencing of the *SMAD6* variants identified in the proband was performed in additional relatives to check if the phenotype segregated with the variant.

### Statistical analysis

We performed burden analyses comparing frequencies of the variants fulfilling the three criteria that were mentioned in “Section Data Analysis and Filtering” between patients and controls. Whereas the Fisher's Exact Test was used to statistically compare variant frequencies in the patient cohort to those in the study control cohort, the Chi-Square Test with Yates' correction was used for the patient-ExAC comparison. No *p*-values were calculated if the number of variants in patients and/or controls was zero. Fisher's Exact statistics were also used to determine if significant variant type enrichment and/or domain clustering of variants occurs in patients. Statistical significance was considered when *p* < 0.05.

## Results

The patient cohort consisted of 441 BAV/TAA patients (75% males and 25% females) with an average age at inclusion of 63.5 ± 14.4 years. For these patients, the most common associated feature was coarctation of the aorta (2.9%, *n* = 13). About 3% (*n* = 14) had other additional findings such as mitral valve prolaps, aortic stenosis, dilated cardiomyopathy, aortic insufficiency, patent ductus arteriosus or intracranial aneurysm. 46.7% (*n* = 206) had a left-right leaflet BAV orientation, 15.9% (*n* = 70) had a right-non-coronary leaflet BAV orientation and for 37.4% (*n* = 165) of the patients the subtype of valve leaflet morphology was not specified. A positive family history was known for 9.3% of the patients, whereas for the remainder the family history was negative or unknown. The study control cohort (*n* = 183) consisted of 58% males and 42% females. The average age at inclusion of this control cohort was 13.1 ± 5.1 years.

Targeted gene panel sequencing reached an overall coverage at 10x of 99.13% of the targeted regions. In total, 169 variants passed our selection criteria in our patient and control group (Supplementary Table 2). Of these, 112 variants were identified in 441 patients. They included 101 missense, 2 nonsense, 2 splice-site, 5 in-frame indel, and 2 frameshift variants. The 183 study controls contained 57 variants including 53 missense, 1 nonsense, 2 splice-site, and 1 frameshift variant. After applying the identical filtering criteria to the ExAC control cohort, 15,660 variants were retained in on average 54,940 individuals: i.e., 14,931 missense, 190 splice-site, 72 nonsense, 10 no-stop, 204 frameshift, and 253 in-frame indel variants.

**Figure 1:**
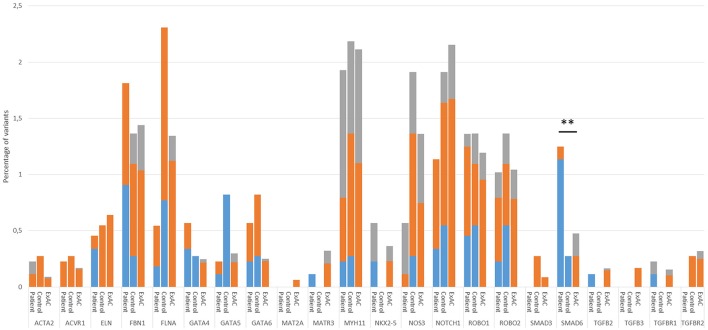
Proportion of variant alleles per gene in the patient group, control group and ExAC cohort. Variants were selected as follows: First, we selected heterozygous coding or splice site variants with an allelic balance between 0.25 and 0.85 (*FLNA* in males: 0.75–1) and a minimum coverage of 10x. Next, we made three variant groups based on their frequency in the ExAC database; that is, variants that are absent from the ExAC control dataset (blue), variants with an ExAC MAF lower than 0.01% (orange) and variants with an ExAC MAF between 0.01% and 0.1% that had a CADD score above 20 (gray). Only statistics of the patient-ExAC comparison are shown (^**^p ≤ 0.01). No statistically significant differences in allele frequencies were observed between our control cohort and the ExAC controls. Abbreviations: ExAC, Exome Aggregation Consortium; MAF, Minor Allele frequency; CADD, Combined Annotation Dependent Depletion.

To validate our control cohort, we compared its variant frequencies for the 22 selected candidate genes to those of the ExAC cohort. No significant differences were observed (Figure 1). We then performed a variant burden analysis equating the numbers of patient variants per gene to the numbers found in the control cohort (Table 2). Results are graphically presented in Figure 1, showing the proportion of variants per gene in the three different cohorts. Although a few genes (e.g., *FLNA*) showed trends toward significance when comparing our study patient and control cohort, we decided to focus on the patient-ExAC comparison because of the larger number of controls in the ExAC cohort and hence, higher power. Only *SMAD6* reached significance (*p* = 0.002) in the patient-ExAC comparison. Remarkably, a protective effect for *NOS3* and *NOTCH1* variants was suggested (*p* = 0.06 and *p* = 0.05, respectively).

**Table 2:** Variant burden comparisons per gene between patients and either study controls or ExAC controls.

**Gene**	**Number of variants in 882 patient alleles**	**Number of variants in 366 control alleles**	**Number of variants in ExAC alleles**	***p*-value patients-controls**	***p*-value patients-ExAC**
*ACTA2*	2	1	109 in 120,631	1.00	0.44
*ACVR1*	2	1	202 in 120,994	1.00	0.98
*ELN*	4	2	728 in 113,954	1.00	0.63
*FBN1*	16	5	1,740 in 120,988	0.81	0.43
*FLNA*	3^*^	6^*^	1,133 in 84,359^*^	**0.03**	0.15
*GATA4*	5	1	260 in 105,980	0.68	0.11
*GATA5*	2	3	259 in 86,819	0.15	0.94
*GATA6*	5	3	240 in 95,775	0.70	0.13
*MAT2A*	0	0	74 in 116,667	/	/
*MATR3*	1	0	382 in 119,089	/	0.43
*MYH11*	17	8	2,513 in 119,001	0.82	0.79
*NKX2-5*	5	0	360 in 98,978	/	0.47
*NOS3*	5	7	1,390 in 102,070	0.05	0.06
*NOTCH1*	10	7	2,181 in 101,245	0.29	0.05
*ROBO1*	12	5	1,354 in 113,390	1.00	0.77
*ROBO2*	9	5	1,245 in 119,282	0.57	0.95
*SMAD3*	0	1	95 in 111,500	/	/
*SMAD6*	11	1	450 in 94,779	0.20	**0.002**
*TGFB2*	1	0	192 in 117,070	/	0.71
*TGFB3*	0	0	205 in 121,315	/	/
*TGFBR1*	2	0	181 in 118,320	/	0.90
*TGFBR2*	0	1	366 in 115,147	/	/

*Variant burden analyses were performed comparing frequencies of the variants fulfilling the three criteria that were mentioned in “Section Data Analysis and Filtering” between patients and controls. Whereas, the Fisher's Exact Test was used to statistically compare variant frequencies in the patient cohort to those in the study control cohort, the Chi-Square Test with Yates' correction was used for the patient-ExAC comparison. No p-values were calculated if the number of variants in patients and/or controls was zero. Statistical significance was considered when p < 0.05. The asterisks denote that in these cases the number of alleles is consistent with the number of X-chromosomes, i.e., 553 patient alleles and 260 control alleles were checked for variants. Statistically significant p-values are represented in bold*.

**Figure 2:**
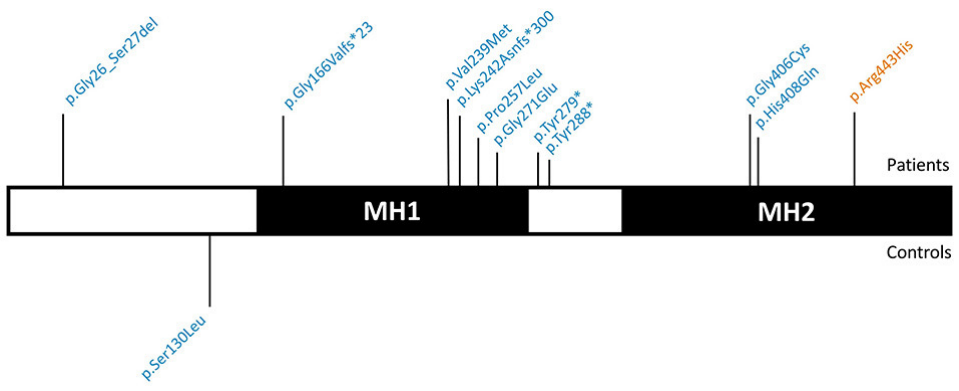
Graphical representation of the identified *SMAD6* variants. SMAD6 has two major protein domains, a DNA-binding MH1 domain and a MH2 domain that interacts with components of the TGF-β and BMP signaling pathways. Variants above the protein have been found in patients, while those below the protein occurred in control individuals. Variants in blue are absent from the ExAC database, variants in orange have an ExAC MAF below 0.01%. Abbreviations: TGF-β, Transforming growth factor-β; BMP, Bone morphogenetic protein; ExAC, Exome Aggregation Consortium; MAF, Minor Allele frequency.

We identified 11 *SMAD6* variants in 441 patients (2.5%). These included two frameshift deletions, two nonsense mutations, one in-frame deletion, and six missense variants (Figure 2). Only a single individual (0.55%) in the study control cohort harbored a *SMAD6* missense variant. The ExAC database harbored 450 *SMAD6* variants in 47,389 individuals (0.9%). Whereas 36.4% (*n* = 4/11) of the *SMAD6* mutations in the patient cohort were loss of function (LOF; frameshift, nonsense or splice site) mutations, truncating *SMAD6* mutations were found in only 4.0% (*n* = 18/450) of the ExAC individuals, demonstrating a clear enrichment in BAV/TAA patients compared to controls (*p* = 0.001).

The *SMAD6* c.726del variant leads to a frameshift (p.Lys242Asnfs^*^300) and a predicted protein with a C-terminal extension due to loss of the intended stop codon. The c.454_461del frameshift variant (p.Gly166Valfs^*^23) causes the introduction of a premature stop codon, most likely resulting in haploinsufficiency due to nonsense-mediated mRNA decay (NMD). Also the two nonsense variants (p.Tyr279^*^ and p.Tyr288^*^) are predicted to lead to NMD. All of the missense variants cluster in the functionally important MH1 and MH2 domains (Makkar et al., 2009) (amino acids 148–275 and 331–496, respectively), which is not the case for the sole missense variant (p.Ser130Leu) found in a control individual (Figure 2). All but one (p.Arg443His) of the identified variants were absent in the ExAC control cohort (v0.3.1; Supplementary Table 2). Moreover, the missense variants in the patient cohort (7/7) are enriched in the MH1 and MH2 domains when compared to ExAC controls (*n* = 228/430; *p* = 0.02).

For two *SMAD6* mutation carriers (P89, p.Gly271Glu; P99, p.Gly166Valfs^*^23), gDNA of family members was available for segregation analysis (Supplementary Figure 1). Although neither of these probands had a documented family history of BAV/TAA, a brother of P89 has been diagnosed with a sinus of Valsalva aneurysm (45 mm) and carried the *SMAD6* mutation. The mutation was also observed in an unaffected daughter (age 28) of the proband (Supplementary Figure 1). Three unaffected siblings at ages 54, 58, and 64 did not carry the mutation. No gDNA was available from a sister of P99 with unspecified aortic valve problems. The p.Gly166Valfs^*^23 mutation was found in an unaffected daughter (age 39) of P99 but was absent in his 39 year-old unaffected son (Supplementary Figure 1).

Intriguingly, two genes (*NOTCH1* and *NOS3*) that previously had been associated with increased BAV risk in humans (Mohamed et al., 1797; Garg et al., 2005; McKellar et al., 2007; Foffa et al., 2013) and/or mice (Lee et al., 2000; Bosse et al., 2013) revealed borderline significance for protection from BAV/TAA (*p* = 0.05 and *p* = 0.06, respectively). Analysis of *NOTCH1* identified 10 variants in patients (2.3%), including two splice-site variants, vs. seven variants (all missense) in controls (3.8%) and 2,181 (4.3%) variants in ExAC. One variant in the patient cohort (c.5167+3_5167+6del) leads to complete loss of the 5' donor splice site of intron 27, predicted to result in skipping of exon 27 (149 bp) and hence a frameshift. For the second variant (p.S784S), the predicted effect on splicing is more ambiguous. If loss of the 5' donor splice site of intron 14 would occur, skipping of exon 14 (146 bp) would again lead to a frameshift event. Unfortunately, cDNA to reliably determine the precise effect of these mutations on splicing is not available. None of the *NOTCH1* variants that we identified in BAV/TAA patients has previously been reported in the literature. We did not observe any variant-domain clustering or significant differences in CADD scores when comparing the patient and control *NOTCH1* variants. Similarly, for *NOS3* a total of five missense variants (1.1%) was found in patients, whereas the control cohort harbored seven variants (3.8%), including one out-of-frame mutation (p.Leu927Hisfs^*^32). In the ExAC control cohort, 1,390 *NOS3* variants (2.7%) were found in 51,035 individuals.

Based on statistical analyses of BAV/TAA heritability and the fact that BAV/TAA shows prominent gender bias, oligogenic inheritance of BAV/TAA is an emerging concept (Andelfinger et al., 2016; Verstraeten et al., 2016). To test for such oligogenic patterns, we determined the number of patients and controls in our study cohort with variants in at least two out of the 22 analyzed genes. In the patient cohort, 10 patients presented with two variants (2.3%), while the control group harbored 7 individuals that carried two variants (3.8%). Based on these data, there is no evidence for a digenic or multigenic model in the analyzed genes (*p* = 0.29).

## Discussion

So far, no gene with a contribution of more than 1% to BAV or BAV/TAA has been identified in humans. Gene identification has been hampered by low penetrance, variable clinical expressivity, the likelihood of BAV-phenocopies within individual families and, most likely, substantial locus heterogeneity (Verstraeten et al., 2016). *NOTCH1* has been suggested as a BAV(/TAA) gene, but does not contribute greatly to disease etiology. About 20 other genes have been associated with BAV in humans and mice (Table 1), but few of them also showed association with TAA. This suggests that whereas some disease genes might be linked to both BAV and TAA, others increase risk for only one of the component phenotypes. In this study, we used a targeted gene panel approach to study the prevalence of mutations in genes that previously have been associated with BAV and/or TAA in people or mice in a cohort of BAV/TAA patients. In total, 22 genes were sequenced in 441 BAV/TAA patients and 183 controls. *SMAD6* was identified as the most important known gene in the etiology of BAV with associated TAA. With 11 mutation-carrying probands, *SMAD6* offers a molecular explanation for 2.5% of our study population. For two of the variants segregation analysis in relatives could be performed, revealing the presence of one of the respective *SMAD6* mutations in a TAA patient and two rather young individuals (age 28 & 39) that might still develop TAA later in life. Four unaffected individuals (age 37, 54, 58, 64) did not carry a *SMAD6* mutation. As two nonsense and two frameshift *SMAD6* variants in our cohort are predicted to lead to haploinsufficiency, LOF is the most likely mechanism. All the patient-specific missense variants (*n* = 7) are in the functionally important MH1 and MH2 domains of SMAD6 (Makkar et al., 2009). LOF missense mutations in *SMAD2* and *SMAD3* causing Loeys-Dietz syndrome, another syndromic TAA form, are also located in the MH1 and MH2 domains (van de Laar et al., 2011; Micha et al., 2015). The MH1 domain of SMAD6 binds DNA (Bai and Cao, 2002), while the MH2 domain interacts with key components of the transforming growth factor (TGF)-β and bone morphogenetic protein (BMP) signaling cascades (Hanyu et al., 2001; Lin et al., 2003; Jung et al., 2013). In 2012, two missense variants in the MH2 domain of SMAD6 were identified in two patients with BAV in association with mild to moderate aortic stenosis (Tan et al., 2012). Interestingly, in our cohort, one *SMAD6* patient (p.Tyr288^*^) presented with coarctation in addition to BAV and TAA. Moreover, mice lacking expression of the murine orthologue of *SMAD6*, i.e., *Madh6*^−/−^ mice, also present with cardiovascular pathologies, including abnormal vascular smooth muscle cell relaxation, thickening of the cardiac valves and misplaced septation and ossification of the outflow tract (OFT) (Galvin et al., 2000). As such, our findings confirm a role for *SMAD6* mutations in the etiology of BAV and expand the spectrum of *SMAD6*-related cardiovascular manifestations with BAV-related TAA.

*SMAD6* is highly expressed in the cardiac valves and OFT of the embryonic heart, in the late-embryonic, and adult vascular endothelium as well as in the vascular smooth muscle cells of the adult aortic root (Galvin et al., 2000; Dickel et al., 2016). Upregulation in response to laminar shear stress has been reported (Topper et al., 1997). *SMAD6* encodes an inhibitory SMAD protein which negatively regulates BMP signaling by binding to BMP type I receptors or by establishing competitive interactions for SMAD4 (Imamura et al., 1997; Hata et al., 1998). In doing so, SMAD1/5/8 phosphorylation and/or nuclear translocation are prevented. Additionally, SMAD6 cooperates with SMURF E3 ubiquitin ligases to prime ubiquitin-mediated proteasomal degradation of BMP receptors and SMAD effector proteins (Murakami et al., 2003), including SMAD1 and 5. BMP signaling has previously been independently implicated in BAV- and TAA-related processes (Cai et al., 2012; Garside et al., 2013). In addition to mediating CNC cell migration into the cardiac cushions and differentiation to smooth muscle cells, BMP signaling promotes endothelial-to-mesenchymal transition and instigates mesenchymal cell invasion (Kaartinen et al., 2004; Garside et al., 2013). While SMAD6 and SMAD7 are thought to have a predominant negative regulatory effect on BMP and TGF-β signaling, respectively, there is strong evidence that this specificity is not absolute and that SMAD6 can directly suppress the TGF-β signaling cascade. Important crosstalk between BMP, TGF-β and NOTCH signaling has been reported (Garside et al., 2013). Many syndromic forms of TAA are caused by mutations in genes encoding effectors or regulators of the TGF-β signaling pathway (including *TGFB2/3, TGFBR1/2, SMAD2/3, SKI*) (Loeys et al., 2005; van de Laar et al., 2011; Boileau et al., 2012; Carmignac et al., 2012; Doyle et al., 2012; Lindsay et al., 2012; Bertoli-Avella et al., 2015; Micha et al., 2015), with increased activity observed in aortic specimens from people and mice with these conditions. An increased prevalence of BAV has been observed in patients carrying mutations in these genes (Table 1). Overall, these results imply that mutations in *SMAD6* likely cause BAV/TAA through impaired negative regulation of BMP and/or TGF-β signaling.

Multiple studies have previously reported a link between *NOTCH1* mutations and BAV (Mohamed et al., 1797; Garg et al., 2005; McKellar et al., 2007; Foffa et al., 2013). In 2005, a nonsense and a frameshift *NOTCH1* mutation were found to segregate with BAV associated with early onset valve calcification in the respective families (Garg et al., 2005). Since the initial report, multiple *NOTCH1*, mostly missense, variants have been associated with BAV, BAV/TAA, aortic valve stenosis, coarctation, and hypoplastic left heart (Mohamed et al., 1797; McKellar et al., 2007; Iascone et al., 2012; Foffa et al., 2013; Freylikhman et al., 2014; Preuss et al., 2016; Irtyuga et al., 2017). In addition to these mutations in association with left-sided heart defects, frameshift and nonsense mutations were also identified in patients with right-sided heart defects affecting the pulmonary valve and conotruncal disease including pulmonary atresia with intact ventricular septum, tetralogy of Fallot, and truncus arteriosus, and other congenital heart diseases, such as anomalous pulmonary venous return, atrial septal defect, and ventricular septal defect (Kerstjens-Frederikse et al., 2016). Mouse models have confirmed a role for Notch1 in the development of the aortic valve and the cardiac OFT (Koenig et al., 2016). Unexpectedly, in our dataset *NOTCH1* did not stand out as a prominent BAV/TAA gene, with the suggestion that *NOTCH1* variants might even be protective. Sample selection bias might contribute to this observation as *NOTCH1* variants appear to associate with early and severe valve calcification and seem to be enriched in families with highly penetrant BAV but far lower penetrance of TAA (Kent et al., 2013). Given that our study did not select for valve calcification and prioritized the BAV/TAA phenotype, it is understandable that *NOTCH1* variants would be underrepresented. It also seems notable that only missense variants were seen in controls, while multiple variants in the patient cohort are predicted to have a more overt impact on protein expression and function.

Similarly, our variant burden test suggested that *NOS3* variants might be protective for BAV/TAA development. NOS3, the endothelial specific nitric oxide (NO) synthase, is important in balancing NO production and in the reduction of oxidative stress (Forstermann and Munzel, 2006). Its role in cardiac development is demonstrated by the formation of BAV in *Nos3*-targeted mice (Table 1). Furthermore, it has already been shown that specific *NOS3* polymorphisms can affect NO production (Oliveira-Paula et al., 2016), and increased NO levels have been found in a MFS mouse model and in *Adamts1-*deficient mice that develop TAA (Oller et al., 2017). Pharmacological inhibition of NOS2 in mice led to a protective effect in aortic aneurysm development (Oller et al., 2017). This supports the importance of NO levels and nitric oxide synthases in aneurysm pathology. The variants in *NOS3* identified in the current study may lead to less active NOS3 and as such may protect against development of aortic aneurysm.

Our study has several methodological limitations: (i) The small number of genes included in our study, as well as the patient cohort size, precludes the ability to detect oligogenic inheritance or gene-gene interactions involved in BAV/TAA. An extended experiment in a larger BAV/TAA cohort, including BAV-related pathways instead of selected genes, could give us more insight regarding how genes work together in BAV and/or TAA development; (ii) The size of the patient and study/ExAC control cohort only allows us to detect BAV/TAA genes with a fairly large contribution (variant burden in patients: ≥3% & ≥2%, respectively); (iii) The control cohort consists of younger, adolescent patients that did not show cardiac complications at the time of investigation but may still develop complications such as TAA later-on in life. Therefore, the ExAC database was used as an additional dataset for allele frequencies in a cohort without gross developmental defects.

Our study specifically assesses the presence of pathogenic variants in BAV-associated genes in a large BAV/TAA cohort. We conclude that *SMAD6* is currently the most important contributor to the genetic architecture of BAV/TAA. More research and larger cohorts will be needed to fully elucidate the genetic architecture of this common but complex cardiovascular pathology.

## Ethics statement

This study was carried out in accordance with written informed consent from all subjects. All subjects gave written informed consent in accordance with the Declaration of Helsinki. The protocol was approved by the Ethics Committee of the Antwerp University Hospital and all participating centers.

## Author contributions

All authors revised the work critically. All authors provided final approval of the version for publication. All authors agreed to be accountable for all aspects of the work and ensure that questions related to the accuracy or integrity of any part of the work are appropriately investigated and resolved. More specific contributions to the work are: EG, AAK, IL, CP, EC, NB, FW, RG, LV, SAM, SM, LM, HB, AF, AM, PE, GA, HD, AV, and BL contributed to conception and design of the work. EG, AAK, IL, EC, MA, NB, GvdB, BW, GV, JM, RZ, DZ, SAM, SM, LM, JV, IV, MW, EM, GG, MN, AK, MK, SS, TD, XJ, JA, PE, AV, and BL contributed to acquisition of the data. EG, AAK, CP, FW, GA, LV, HD, AV, and BL contributed to analysis of the data. EG, IL, CP, EC, MA, FW, RG, RZ, DZ, SAM, SM, LM, HB, AF, AM, LV, JV, IV, MW, EM, GG, MN, AK, MK, SS, TD, XJ, JA, PE, HD, AV, and BL contributed to interpretation of the data.

### Conflict of interest statement

The authors declare that the research was conducted in the absence of any commercial or financial relationships that could be construed as a potential conflict of interest.
