# Corrigendum: Candidate Gene Resequencing in a Large Bicuspid Aortic Valve-Associated Thoracic Aortic Aneurysm Cohort: *SMAD6* as an Important Contributor

**DOI:** 10.3389/fphys.2017.00730

**Published:** 2017-09-25

**Authors:** Elisabeth Gillis, Ajay A. Kumar, Ilse Luyckx, Christoph Preuss, Elyssa Cannaerts, Gerarda van de Beek, Björn Wieschendorf, Maaike Alaerts, Nikhita Bolar, Geert Vandeweyer, Josephina Meester, Florian Wünnemann, Russell A. Gould, Rustam Zhurayev, Dmytro Zerbino, Salah A. Mohamed, Seema Mital, Luc Mertens, Hanna M. Björck, Anders Franco-Cereceda, Andrew S. McCallion, Lut Van Laer, Judith M. A. Verhagen, Ingrid M. B. H. van de Laar, Marja W. Wessels, Emmanuel Messas, Guillaume Goudot, Michaela Nemcikova, Alice Krebsova, Marlies Kempers, Simone Salemink, Toon Duijnhouwer, Xavier Jeunemaitre, Juliette Albuisson, Per Eriksson, Gregor Andelfinger, Harry C. Dietz, Aline Verstraeten, Bart L. Loeys, Mibava Leducq Consortium

**Affiliations:** ^1^Faculty of Medicine and Health Sciences, Center of Medical Genetics, University of Antwerp and Antwerp University Hospital Antwerp, Belgium; ^2^Cardiovascular Genetics, Department of Pediatrics, CHU Sainte-Justine, Université de Montreal Montreal, QC, Canada; ^3^Department of Cardiac and Thoracic Vascular Surgery, University Hospital Schleswig-Holstein Lübeck, Germany; ^4^McKusick-Nathans Institute of Genetic Medicine, Johns Hopkins University School of Medicine Baltimore, MD, United States; ^5^Department of Clinical Pathology, Lviv National Medical University after Danylo Halytsky Lviv, Ukraine; ^6^Cardiovascular Research, SickKids University Hospital Toronto, ON, Canada; ^7^Cardiovascular Medicine Unit, Department of Medicine, Karolinska Institute Stockholm, Sweden; ^8^Cardiothoracic Surgery Unit, Department of Molecular Medicine and Surgery, Karolinska Institute Stockholm, Sweden; ^9^Department of Clinical Genetics, Erasmus University Medical Center Rotterdam, Netherlands; ^10^Assistance Publique–Hôpitaux de Paris, Hôpital Européen Georges Pompidou; Université Paris Descartes, Paris Sorbonne Cité; Institut National de la Santé et de la Recherche Médicale, UMRS Paris, France; ^11^Department of Biology and Medical Genetics, 2nd Faculty of Medicine-Charles University and Motol University Hospital Prague, Czechia; ^12^Institute of Clinical and Experimental Medicine Prague, Czechia; ^13^Department of Human Genetics, Radboud University Medical Centre Nijmegen, Netherlands; ^14^Howard Hughes Medical Institute Baltimore, MD, United States

**Keywords:** bicuspid aortic valve, thoracic aortic aneurysm, SMAD6, targeted gene panel, variant burden test

In the original article, we noted two mutation annotation errors. The correction of these two mistakes does not change the scientific conclusions in any way. The authors apologize for these nomenclature errors. Please find below the corrected annotations of those two mutations:

(1) The correct RNA and protein annotations of the *SMAD6* variant in P99 are c.455_461del and p.Pro152Profs^*^27, and not c.454_461del and p.Gly166Valfs^*^23.

(2) The correct RNA and protein annotations of the *SMAD6* variant in P128 are c.74_79del and p.Ser27_Gly28del, and not c.73_79del and p.Gly26_Ser27del.

As a consequence, a correction has been made to RESULTS, Paragraphs 5 and 6:

The *SMAD6* c.726del variant leads to a frameshift (p.Lys242Asnfs^*^300) and a predicted protein with a C-terminal extension due to loss of the intended stop codon. The c.455_461del frameshift variant (p.Pro152Profs^*^27) causes the introduction of a premature stop codon, most likely resulting in haploinsufficiency due to nonsense-mediated mRNA decay (NMD). Also the two nonsense variants (p.Tyr279^*^ and p.Tyr288^*^) are predicted to lead to NMD. All of the missense variants cluster in the functionally important MH1 and MH2 domains (Makkar et al., 2009) (amino acids 148–275 and 331–496, respectively), which is not the case for the sole missense variant (p.Ser130Leu) found in a control individual (Figure 2). All but one (p.Arg443His) of the identified variants were absent in the ExAC control cohort (v0.3.1; Supplementary Table 2). Moreover, the missense variants in the patient cohort (7/7) are enriched in the MH1 and MH2 domains when compared to ExAC controls (*n* = 228/430; *p* = 0.02).

**Figure 2:**
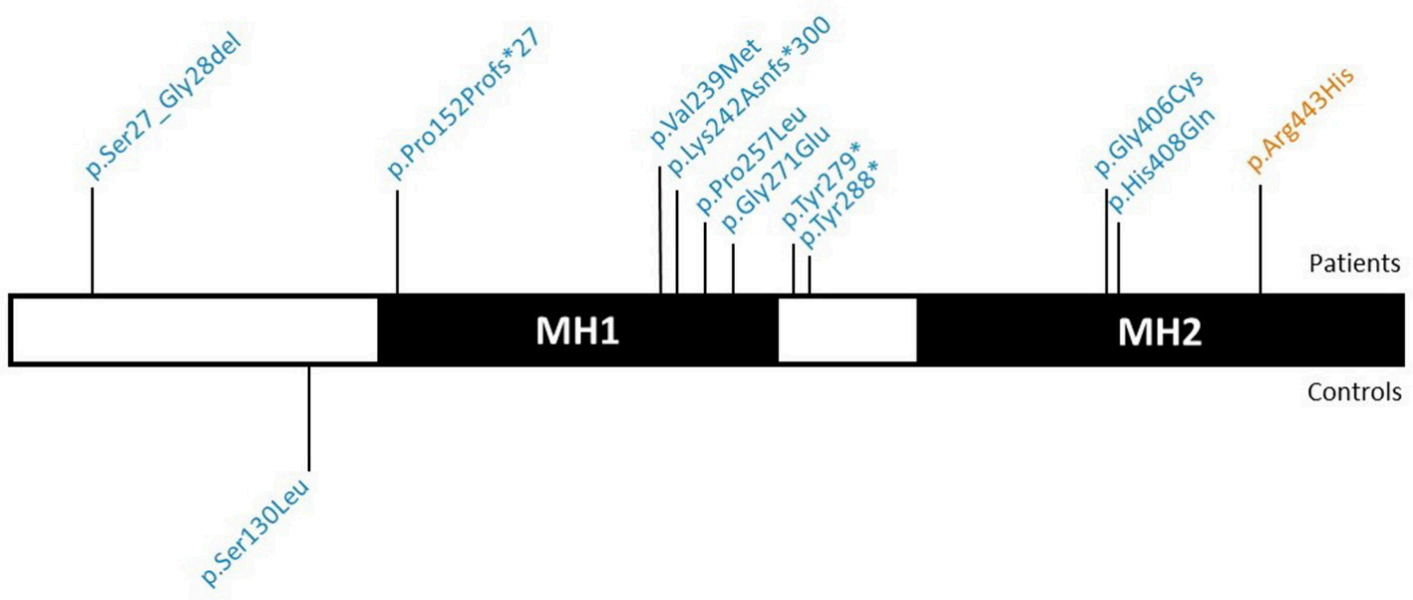
Graphical representation of the identified *SMAD6* variants. SMAD6 has two major protein domains, a DNA-binding MH1 domain and a MH2 domain that interacts with components of the TGF-β and BMP signaling pathways. Variants above the protein have been found in patients, while those below the protein occurred in control individuals. Variants in blue are absent from the ExAC database, variants in orange have an ExAC MAF below 0.01%. TGF-β, Transforming growth factor-β; BMP, Bone morphogenetic protein; ExAC, Exome Aggregation Consortium; MAF, Minor Allele frequency.

For two SMAD6 mutation carriers (P89, p.Gly271Glu; P99, p.Pro152Profs^*^27), gDNA of family members was available for segregation analysis (Supplementary Figure 1). Although neither of these probands had a documented family history of BAV/TAA, a brother of P89 has been diagnosed with a sinus of Valsalva aneurysm (45 mm) and carried the SMAD6 mutation. The mutation was also observed in an unaffected daughter (age 28) of the proband (Supplementary Figure 1). Three unaffected siblings at ages 54, 58, and 64 did not carry the mutation. No gDNA was available from a sister of P99 with unspecified aortic valve problems. The p.Pro152Profs^*^27 mutation was found in an unaffected daughter (age 39) of P99 but was absent in his 37 year-old unaffected son (Supplementary Figure 1).

We also provide a corrected Figure 2 and Supplementary Table 2.

## Conflict of interest statement

The authors declare that the research was conducted in the absence of any commercial or financial relationships that could be construed as a potential conflict of interest.
