# Supplementary material for: Candidate Gene Resequencing in a Large Bicuspid Aortic Valve-Associated Thoracic Aortic Aneurysm Cohort: SMAD6 as an Important Contributor
Source: Front Physiol. 2017 Jun 13;8:400. doi: 10.3389/fphys.2017.00400 (PMC5469151; doi:10.3389/fphys.2017.00400)
Supplement: Supplementary file 1 [file Table1.DOCX]

Supplementary Material

Candidate Gene Resequencing In A Large Bicuspid Aortic Valve-Associated Thoracic Aortic Aneurysm Cohort: SMAD6 As An Important Contributor

**E. Gillis^1#^, A. Kumar^1#^, I. Luyckx^1^, C. Preuss^2^, E. Cannaerts^1^, G. van de Beek^1^, B. Wieschendorf^1,3^, M. Alaerts^1^, N. Bolar^1^, G. Vandeweyer^1^, J. Meester^1^, F. Wünnemann^2^, R.A. Gould^4^, R. Zhurayev^5^, D. Zerbino^5^, S.A. Mohamed^3^, S. Mital^6^, L. Mertens^6^, H.M. Björck^7^, A. Franco-Cereceda^8^, A. S. Mc Callion^4^, L. Van Laer^1^, J.M.A. Verhagen^9^, I.M.B.H. van de Laar^9^, M.W. Wessels^9^, E. Messas^10^, G. Goudot^10^, M. Nemcikova^11^, A. Krebsova^12^, M. Kempers^13^, S. Salemink^13^, T. Duijnhouwer^13^, X. Jeunemaitre^10^, J. Albuisson^10^, P. Eriksson^7^, G. Andelfinger^2^, H. Dietz^4,14^, A. Verstraeten^1^, B.L. Loeys^1,13*^, Mibava Leducq Consortium.**

*** Correspondence:** Prof. Dr. Bart Loeys: Bart.Loeys@uantwerpen.be

# Supplementary Tables

**Supplementary Table 1.** Patient cohort overview

| **Centre** | **City, Country** | **#** |
| --- | --- | --- |
| Radboud University Medical Centre | Nijmegen, the Netherlands | 27 |
| APHP-Hˆopital Europ´een Georges Pompidou | Paris, France | 59 |
| Erasmus University Medical Center | Rotterdam, the Netherlands | 30 |
| University of Luebeck | Luebeck, Germany | 87 |
| Institute for Clinical and Ex- perimental Medicine | Prague, Czech Republic | 16 |
| Sickkids Hospital | Toronto, Canada | 62 |
| Karolinska University Hospital, Karolinska Institutet | Stockholm, Sweden | 156 |
| Lviv National Medical University after Danylo Halytsky | Lviv, Ukraine | 4 |
| Total |  | 441 |
