# Supplementary material for: Candidate Gene Resequencing in a Large Bicuspid Aortic Valve-Associated Thoracic Aortic Aneurysm Cohort: SMAD6 as an Important Contributor
Source: Front Physiol. 2017 Jun 13;8:400. doi: 10.3389/fphys.2017.00400 (PMC5469151; doi:10.3389/fphys.2017.00400)
Supplement: Supplementary file 2 [file Table2.DOCX]

Supplementary Material

Candidate Gene Resequencing In A Large Bicuspid Aortic Valve-Associated Thoracic Aortic Aneurysm Cohort: SMAD6 As An Important Contributor

**E. Gillis^1#^, A. Kumar^1#^, I. Luyckx^1^, C. Preuss^2^, E. Cannaerts^1^, G. van de Beek^1^, B. Wieschendorf^1,3^, M. Alaerts^1^, N. Bolar^1^, G. Vandeweyer^1^, J. Meester^1^, F. Wünnemann^2^, R.A. Gould^4^, R. Zhurayev^5^, D. Zerbino^5^, S.A. Mohamed^3^, S. Mital^6^, L. Mertens^6^, H.M. Björck^7^, A. Franco-Cereceda^8^, A. S. Mc Callion^4^, L. Van Laer^1^, J.M.A. Verhagen^9^, I.M.B.H. van de Laar^9^, M.W. Wessels^9^, E. Messas^10^, G. Goudot^10^, M. Nemcikova^11^, A. Krebsova^12^, M. Kempers^13^, S. Salemink^13^, T. Duijnhouwer^13^, X. Jeunemaitre^10^, J. Albuisson^10^, P. Eriksson^7^, G. Andelfinger^2^, H. Dietz^4,14^, A. Verstraeten^1^, B.L. Loeys^1,13*^, Mibava Leducq Consortium.**

*** Correspondence:** Prof. Dr. Bart Loeys: Bart.Loeys@uantwerpen.be

# Supplementary Figures and Tables

## Supplementary Tables

**Supplementary Table 2.** Overview of the identified variants in the genes from the targeted gene panel and their phenotypic data.

| Gene | ID | Sex | BAV type | Nucleotide change | Protein change | Classifi-cation | MAF ExAC | CADD score |
| --- | --- | --- | --- | --- | --- | --- | --- | --- |
| *ACTA2* | P11 | M | NA | c.678A>C | p.Glu226Asp | Missense | 1/121150 | 15.8 |
|  | P281 | M | L-R | c.977C>A | p.Thr326Asn | Missense | 16/121284 | 21.2 |
|  | C102 | F | TAV | c.959C>T | p.Thr320Met | Missense | 7/121328 | 19.1 |
|  |  |  |  |  |  |  |  |  |
| *ACVR1* | P25 | F | NA | c.636G>C | p.Glu212Asp | Missense | 5/121074 | 18.7 |
|  | P286 | M | R-N | c.718C>T | p.Arg240Cys | Missense | 1/121392 | 19.8 |
|  | C172 | M | TAV | c.1394C>T | p.Pro465Leu | Missense | 7/121410 | 23.9 |
|  |  |  |  |  |  |  |  |  |
| *ELN* | P311 | F | L-R | c.1114_1125del | p.Ala372_Lys375del | In-frame deletion | Absent | / |
|  | P349 | M | NA | c.1114_1125del | p.Ala372_Lys375del | In-frame deletion | Absent | / |
|  | P179 | F | R-N | c.1421_1422insTC  CTGGTGTCG  GCGTGGC | p.Pro475_Ala480dup | In-frame duplication | Absent | / |
|  | P381 | F | L-R | c.1909G>A | p.Ala637Thr | Missense | 1/17222 | 12.2 |
|  | C165 | M | TAV | c.1021G>A | p.Val341Ile | Missense | 4/121176 | 0 |
|  | C95 | F | TAV | c.1883G>C† | p.Gly628Ala | Missense | 6/111550 | 11.8 |
|  |  |  |  |  |  |  |  |  |
| *FBN1* | P117 | F | NA | c.1158C>G | p.Asn386Lys | Missense | 8/119050 | 13.8 |
|  | P126 | M | NA | c.1472T>C | p.Val491Ala | Missense | Absent | 16 |
|  | P227 | F | L-R | c.1651G>A | p.Gly551Ser | Missense | 1/121162 | 36 |
|  | P431 | M | L-R | c.2115G>A | p.Ala705Ala | Splice site | Absent | 11.5 |
|  | P287 | M | R-N | c.2315A>G | p.Asn772Ser | Missense | Absent | 23.7 |
|  | P174 | M | R-N | c.3142A>G | p.Ile1048Val | Missense | Absent | 18.8 |
|  | P28 | M | NA | c.3382G>A | p.Val1128Ile | Missense | Absent | 8.4 |
|  | P132 | M | NA | c.3797A>T | p.Tyr1266Phe | Missense | 12/121316 | 12.5 |
|  | P137 | F | NA | c.4340T>C | p.Ile1447Thr | Missense | Absent | 19 |
|  | P196 | M | NA | c.4609G>T | p.Asp1537Tyr | Missense | Absent | 22.1 |
|  | P426 | M | NA | c.4727T>C | p.Met1576Thr | Missense | 10/121398 | 6.5 |
|  | P117 | F | NA | c.5123G>A | p.Gly1708Glu | Missense | 1/121388 | 23.7 |
|  | P85 | F | NA | c.6595G>A | p.Gly2199Ser | Missense | 1/121280 | 36 |
|  | P332 | M | L-R | c.6783A>C | p.Lys2261Asn | Missense | 4/121108 | 16 |
|  | P150 | F | R-N | c.7846A>G | p.Ile2616Val | Missense | 8/121320 | 3 |
|  | P41 | M | NA | c.8232G>C | p.Gln2744His | Missense | Absent | 8.4 |
|  | C32 | M | TAV | c.185G>A | p.Arg62His | Missense | 7/119890 | 17 |
|  | C28 | M | TAV | c.716T>C | p.Ile239Thr | Missense | 2/121008 | 13.8 |
|  | C55 | F | TAV | c.1118C>T | p.Ala373Val | Missense | 1/121226 | 9.2 |
|  | C29 | M | TAV | c.6163+2dupT | / | Splice site | Absent | / |
|  | C167 | M | TAV | c.6832C>G | p.Pro2278Ala | Missense | 19/121266 | 25.7 |
|  |  |  |  |  |  |  |  |  |
| *FLNA* | P209 | F | L-R | c.2906T>C | p.Leu969Pro | Missense | 4/86745 | 20.3 |
|  | P434 | M | R-N | c.5908G>A | p.Asp1970Asn | Missense | Absent | 23.5 |
|  | P51 | M | NA | c.7172G>A | p.Arg2391His | Missense | 7/86932 | 18.2 |
|  | C160 | F | TAV | c.C901C>T | p.Arg301Trp | Missense | 6/85694 | 11.6 |
|  | C132 | F | TAV | c.1270A>G | p.Met424Val | Missense | 2/85774 | 0.1 |
|  | C136 | F | TAV | c.2738G>C | p.Gly913Ala | Missense | 2/86788 | 11.8 |
|  | C55 | F | TAV | c.3346G>C | p.Asp1116His | Missense | Absent | 19.4 |
|  | C81 | M | TAV | c.4520A>G | p.Gln1507Arg | Missense | Absent | 18.2 |
|  | C167 | M | TAV | c.4711G>A | p.Asp1571Asn | Missense | 2/82272 | 35 |
|  |  |  |  |  |  |  |  |  |
| *GATA4* | P339 | M | L-R | c.142G>T | p.Val48Leu | Missense | Absent | 0.2 |
|  | P370 | M | NA | c.173G>T | p.Gly58Val | Missense | Absent | 10.2 |
|  | P177 | M | L-R | c.611A>G | p.Asn204Ser | Missense | Absent | 5 |
|  | P29 | M | NA | c.939G>T | p.Glu313Asp | Missense | 8/120012 | 16.5 |
|  | P30 | M | NA | c.939G>T | p.Glu313Asp | Missense | 8/120012 | 16.5 |
|  | C155 | M | TAV | c.175G>T | p.Ala59Ser | Missense | Absent | 0 |
|  |  |  |  |  |  |  |  |  |
| *GATA5* | P438 | M | L-R | c.472C>T | p.Pro158Ser | Missense | Absent | 15.4 |
|  | P98 | F | NA | c.616G>A | p.Gly206Ser | Missense | 0.00003 | 36 |
|  | C161 | F | TAV | c.287C>G | p.Ala96Gly | Missense | Absent | 3.9 |
|  | C104 | M | TAV | c.395G>A | p.Arg132Gln | Missense | Absent | 16.1 |
|  | C152 | F | TAV | c.1153G>T | p.Ala385Ser | Missense | Absent | 0 |
|  |  |  |  |  |  |  |  |  |
| *GATA6* | P138 | M | NA | c.148G>A | p.Gly50Arg | Missense | 1/95966 | 18.8 |
|  | P395 | F | NA | c.271C>T | p.Pro91Ser | Missense | 6/73362 | 12 |
|  | P133 | M | NA | c.706G>T | p.Gly236Cys | Missense | Absent | 11.5 |
|  | P289 | M | L-R | c.968_976delACCACCACC | p.His324_His326del | In-frame deletion | Absent | 0 |
|  | P98 | F | NA | c.1555A>G | p.Thr519Ala | Missense | 3/121370 | 12.4 |
|  | C166 | M | TAV | c.89G>A | p.Arg30Gln | Missense | 1/106184 | 16.4 |
|  | C94 | M | TAV | c.352C>T | p.Leu118Phe | Missense | 6/103504 | 14.7 |
|  | C130 | F | TAV | c.727G>T | p.Gly243Cys | Missense | Absent | 9.1 |
|  |  |  |  |  |  |  |  |  |
| *MAT2A* |  |  |  |  |  |  |  |  |
|  |  |  |  |  |  |  |  |  |
| *MATR3* | P255 | M | L-R | c.35G>A | p.Arg12Lys | Missense | Absent | 16.4 |
|  |  |  |  |  |  |  |  |  |
| *MYH11* | P377 | F | L-R | c.2026C>T | p.Arg676Cys | Missense | 70/121144 | 21.5 |
|  | P167 | F | R-N | c.2026C>T | p.Arg676Cys | Missense | 70/121144 | 21.5 |
|  | P181 | M | R-N | c.2026C>T | p.Arg676Cys | Missense | 70/121144 | 21.5 |
|  | P252 | M | L-R | c.2026C>T | p.Arg676Cys | Missense | 70/121144 | 21.5 |
|  | P372 | M | L-R | c.2981T>A | p.Ile994Asn | Missense | Absent | 12.4 |
|  | P153 | M | L-R | c.3784A>G | p.Lys1262Glu | Missense | 1/121400 | 29.4 |
|  | P252 | M | L-R | c.3826A>G | p.Ser1276Gly | Missense | 6/121368 | 15.4 |
|  | P160 | M | L-R | c.3848C>T | p.Ala1283Val | Missense | 6/121224 | 7.2 |
|  | P7 | F | NA | c.3917C>A | p.Ala1306Asp | Missense | Absent | 19.3 |
|  | P314 | F | L-R | c.4531C>T | p.Arg1511Trp | Missense | 7/121404 | 19.6 |
|  | P79 | F | L-R | c.4624C>T | p.Arg1542Trp | Missense | 15/121346 | 21.1 |
|  | P57 | F | NA | c.4694C>T | p.Thr1565Met | Missense | 90/121408 | 23.2 |
|  | P223 | M | R-N | c.4694C>T | p.Thr1565Met | Missense | 90/121408 | 23.2 |
|  | P88 | M | NA | c.4681G>A | p.Ala1568Thr | Missense | 15/121408 | 34 |
|  | P64 | M | L-R | c.5247G>C | p.Glu1749Asp | Missense | 66/90680 | 21.3 |
|  | P341 | M | L-R | c.5294G>A | p.Arg1765Gln | Missense | 25/116672 | 33 |
|  | P391 | M | L-R | c.5687C>T | p.Ala1896Val | Missense | 6/121208 | 25.9 |
|  | C110 | F | TAV | c.33G>T | p.Glu11Asp | Missense | 7/121350 | 11.6 |
|  | C23 | F | TAV | c.1223T>C | p.Ile408Thr | Missense | 1/121412 | 21 |
|  | C40 | F | TAV | c.1934C>T | p.Ser645Leu | Missense | 17/106978 | 24.8 |
|  | C81 | M | TAV | c.1934C>T | p.Ser645Leu | Missense | 17/106978 | 24.8 |
|  | C173 | M | TAV | c.3430G>T | p.Ala1144Ser | Missense | 1/121412 | 18.6 |
|  | C95 | F | TAV | c.3583C>T | p.Arg1195Trp | Missense | 20/121406 | 24.1 |
|  | C34 | F | TAV | c.4599C>T | p.Asn1533Asn | Splice site | Absent | 8.5 |
|  | C143 | M | TAV | c.5687C>T | p.Ala1896Val | Missense | 6/121208 | 25.9 |
|  |  |  |  |  |  |  |  |  |
| *NKX2-5* | P30 | M | NA | c.61G>C | p.Glu21Gln | Missense | 92/114290 | 22.9 |
|  | P352 | M | L-R | c.61G>C | p.Glu21Gln | Missense | 92/114290 | 22.9 |
|  | P273 | M | L-R | c.89C>A | p.Ala30Asp | Missense | Absent | 16.1 |
|  | P157 | F | R-N | c.358G>T | p.Val120Leu | Missense | Absent | 4.8 |
|  | P257 | F | NA | c.650G>A | p.Arg217Lys | Missense | 21/92066 | 24.6 |
|  |  |  |  |  |  |  |  |  |
| *NOS3* | P343 | M | L-R | c.466G>A | p.Glu156Lys | Missense | 26/94176 | 36 |
|  | P336 | M | R-N | c.668A>G | p.Asn223Ser | Missense | 2/112186 | 22.6 |
|  | P65 | M | L-R | c.1267G>A | p.Ala423Thr | Missense | 73/120868 | 25.1 |
|  | P175 | F | R-N | c.2457C>G | p.Asp819Glu | Missense | 5/11892 | 23.7 |
|  | P382 | M | NA | c.2642C>T | p.Ala881Val | Missense | 14/119328 | 22.4 |
|  | C19 | M | TAV | c.466G>A | p.Glu156Lys | Missense | 26/94176 | 36 |
|  | C6 | M | TAV | c.638A>G | p.Asn213Ser | Missense | Absent | 13.7 |
|  | C68 | M | TAV | c.1267G>A | p.Ala423Thr | Missense | 73/120868 | 25.1 |
|  | C85 | M | TAV | c.2471C>T | p.Thr824Ile | Missense | 1/11184 | 12.7 |
|  | C152 | F | TAV | c.2546G>A | p.Arg849Gln | Missense | 1/115870 | 36 |
|  | C151 | M | TAV | c.2776_2776delinsCCA | p.Leu927Hisfs*32 | Frameshift | 1/84330 | / |
|  | C182 | F | TAV | c.3589G>A | p.Gly1197Ser | Missense | 3/110874 | 7.3 |
|  |  |  |  |  |  |  |  |  |
| *NOTCH1* | P113 | M | NA | c.983C>G | p.Thr328Ser | Missense | 1/119274 | 18.3 |
|  | P373 | F | L-R | c.1951G>A | p.Asp651Asn | Missense | Absent | 14.7 |
|  | P344 | F | L-R | c.2352C>T | p.Ser784Ser | Splice site | 7/118434 | 9.1 |
|  | P423 | M | L-R | c.4013C>T | p.Ala1338Val | Missense | Absent | 19.1 |
|  | P155 | M | R-N | c.4021G>A | p.Glu1341Lys | Missense | 3/66948 | 7.7 |
|  | P128 | M | NA | c.5047C>T | p.Arg1683Trp | Missense | 1/119254 | 29.4 |
|  | P202 | F | L-R | c.5167+3_5167+6del |  | Splice site | Absent | / |
|  | P134 | M | NA | c.5248G>A | p.Val1750Met | Missense | 3/100758 | 17.3 |
|  | P420 | M | L-R | c.5414T>C | p.Leu1805Pro | Missense | 3/119106 | 22.1 |
|  | P106 | M | NA | c.6413C>T | p.Pro2138Leu | Missense | 1/114110 | 3.6 |
|  | C103 | M | TAV | c.121A>G | p.Asn41Asp | Missense | Absent | 12.8 |
|  | C45 | M | TAV | c.800A>G | p.Lys267Arg | Missense | 1/119480 | 11.1 |
|  | C162 | M | TAV | c.2003C>T | p.Pro668Leu | Missense | 4/118872 | 17.7 |
|  | C158 | M | TAV | c.2003C>T | p.Pro668Leu | Missense | 4/118872 | 17.7 |
|  | C54 | M | TAV | c.5273G>A | p.Arg1758His | Missense | 13/107714 | 27.1 |
|  | C101 | M | TAV | c.7361A>G | p.His2454Arg | Missense | Absent | 11.9 |
|  | C49 | F | TAV | c.7372C>A | p.Pro2458Thr | Missense | 2/78680 | 12.9 |
|  |  |  |  |  |  |  |  |  |
| *ROBO1* | P344 | F | L-R | c.153C>A | p.Asp51Glu | Missense | Absent | 22 |
|  | P252 | M | L-R | c.497C>G | p.Ala166Gly | Missense | Absent | 21.9 |
|  | P5 | M | NA | c.703G>A | p.Ala235Thr | Missense | 5/118284 | 34 |
|  | P432 | M | L-R | c.818T>C | p.Val273Ala | Missense | 15/120622 | 23.1 |
|  | P374 | M | R-N | c.979T>C | p.Ser327Pro | Missense | 23/120206 | 21.2 |
|  | P328 | M | NA | c.979T>C | p.Ser327Pro | Missense | 23/120206 | 21.2 |
|  | P69 | F | R-N | c.1432G>C | p.Ala478Pro | Missense | Absent | 33 |
|  | P5 | M | NA | c.1616A>G | p.Tyr539Cys | Missense | 5/120482 | 15.9 |
|  | P191 | M | NA | c.3007G>A | p.Asp1003Asn | Missense | 3/120414 | 19 |
|  | P384 | M | L-R | c.3259A>C | p.Met1087Leu | Missense | 8/120756 | 7.1 |
|  | P348 | M | NA | c.3472A>G | p.Ser1158Gly | Missense | Absent | 16.1 |
|  | P34 | M | NA | c.4821G>A | p.Met1607Ile | Missense | 2/120590 | 19.4 |
|  | C99 | M | TAV | c.394A>G | p.Ile132Val | Missense | Absent | 18.9 |
|  | C105 | M | TAV | c.508G>A | p.Asp170Asn | Missense | Absent | 24.3 |
|  | C145 | M | TAV | c.818T>C | p.Val273Ala | Missense | 15/120622 | 23.1 |
|  | C60 | M | TAV | c.2987C>T | p.Thr996Met | Missense | 2/120118 | 17.8 |
|  | C158 | M | TAV | c.3259A>C | p.Met1087Leu | Missense | 8/120756 | 7.1 |
|  |  |  |  |  |  |  |  |  |
| *ROBO2* | P48 | F | NA | c.1238C>T | p.Thr413Ile | Missense | 10/120348 | 14.2 |
|  | P423 | M | L-R | c.1859C>T | p.Pro620Leu | Missense | Absent | 21.4 |
|  | P364 | F | NA | c.2018G>T | p.Arg673Leu | Missense | Absent | 31 |
|  | P432 | M | L-R | c.2897C>T | p.Thr966Met | Missense | 5/120752 | 21.3 |
|  | P164 | M | L-R | c.3229C>G | p.Pro1077Ala | Missense | 6/120516 | 13.1 |
|  | P29 | M | NA | c.3230C>A | p.Pro1077His | Missense | 2/120524 | 17.2 |
|  | P180 | F | L-R | c.3857G>T | p.Arg1286Leu | Missense | 67/120712 | 27 |
|  | P250 | F | L-R | c.3857G>T | p.Arg1286Leu | Missense | 67/120712 | 27 |
|  | P168 | M | R-N | c.4063C>T | p.Arg1355Cys | Missense | 3/120728 | 15 |
|  | C90 | F | TAV | c.406C>T | p.Arg136* | Nonsense | Absent | 37 |
|  | C20 | M | TAV | c.2018G>A | p.Arg673His | Missense | 16/120314 | 32 |
|  | C125 | F | TAV | c.2390G>A | p.Arg797Gln | Missense | 6/120660 | 21 |
|  | C74 | M | TAV | c.2902C>G | p.Leu968Val | Missense | Absent | 17.7 |
|  | C5 | F | TAV | c.3230C>A | p.Pro1077His | Missense | 2/120524 | 17.2 |
|  |  |  |  |  |  |  |  |  |
| *SMAD3* | C72 | M | TAV | c.448T>C | p.Phe150Leu | Missense | 2/121372 | 11.9 |
|  |  |  |  |  |  |  |  |  |
| *SMAD6* | P128 | M | NA | c.73_79del | p.Gly26_Ser27del | In-frame deletion | Absent | / |
|  | P99 | M | NA | c.454_461del | p.Gly166Valfs*23 | Frameshift deletion | Absent | / |
|  | P94 | M | NA | c.715G>A | p.Val239Met | Missense | Absent | 24.4 |
|  | P231 | M | L-R | c.726del | p.Lys242Asnfs*300 | Frameshift deletion | Absent | / |
|  | P12 | F | NA | c.770C>T | p.Pro257Leu | Missense | Absent | 16 |
|  | P89 | M | NA | c.812G>A | p.Gly271Glu | Missense | Absent | 23.6 |
|  | P308 | F | L-R | c.837C>A | p.Tyr279* | Nonsense | Absent | 38 |
|  | P180 | F | L-R | c.864C>G | p.Tyr288* | Nonsense | Absent | 38 |
|  | P367 | M | R-N | c.1216G>T | p.Gly406Cys | Missense | Absent | 19.6 |
|  | P67 | F | R-N | c.1224C>G | p.His408Gln | Missense | Absent | 18.3 |
|  | P201 | M | NA | c.1328G>A | p.Arg443His | Missense | 1/106314 | 23.7 |
|  | C148 | M | TAV | c.389C>T | p.Ser130Leu | Missense | Absent | 12.8 |
|  |  |  |  |  |  |  |  |  |
| *TGFB2* | P133 | M | NA | c.1048C>T | p.Leu350Phe | Missense | Absent | 18.8 |
|  |  |  |  |  |  |  |  |  |
| *TGFB3* |  |  |  |  |  |  |  |  |
|  |  |  |  |  |  |  |  |  |
| *TGFBR1* | P334 | M | L-R | c.119T>A | p.Leu40His | Missense | Absent | 9.5 |
|  | P105 | M | NA | c.926C>T | p.Thr309Met | Missense | 2/121364 | 24.9 |
|  |  |  |  |  |  |  |  |  |
| *TGFBR2* | C72 | M | TAV | c.1090C>T | p.Arg364Trp | Missense | 6/120734 | 14.7 |

The patient ID prefix denotes variants identified in patients (P) and controls (C). Bicuspid aortic valve (BAV) subtypes can be left-right (L-R), right-non-coronary (R-N), or unkown (NA). Controls have a tricuspid aortic valve (TAV). Used RefSeq transcripts (except †NM_001278939): NM_001613 (*ACTA2*), NM_001105 (*ACVR1*), NM_000501 (*ELN*), NM_000138 (*FBN1*), NM_212482 (*FN1*), NM_002052 (*GATA4*), NM_080473 (*GATA5*), NM_005257 (*GATA6*), NM_018834 (*MATR3*), NM_001040113 (*MYH11*), NM_172387 (*NFTAC1*), NM_004387 (*NKX2-5*), NM_000603 (*NOS3*), NM_017617 (*NOTCH1*), NM_002941 (*ROBO1*), NM_002942 (*ROBO2*), NM_005902 (*SMAD3*), NM_005585 (*SMAD6*), NM_001135599 (*TGFB2*), NM_004612 (*TGFBR1*), NM_001024847 (TGFBR2).
