# Supplementary figures and images for: Candidate Gene Resequencing in a Large Bicuspid Aortic Valve-Associated Thoracic Aortic Aneurysm Cohort: SMAD6 as an Important Contributor
Source: Front Physiol. 2017 Jun 13;8:400. doi: 10.3389/fphys.2017.00400 (PMC5469151; doi:10.3389/fphys.2017.00400)

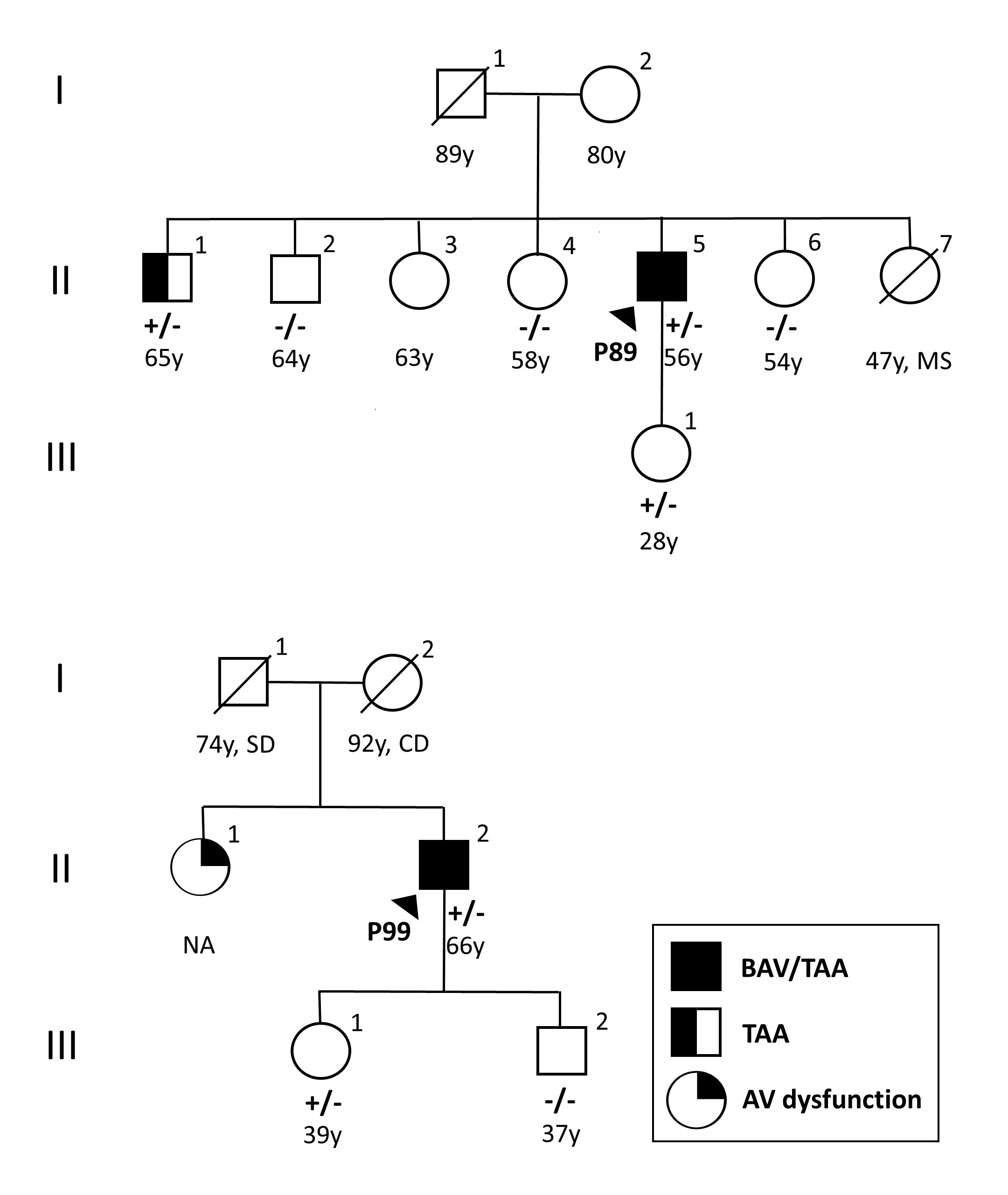

Supplement: Supplementary file 3 [file Image1.JPEG]
